# Efficacy Study of Folic Acid Supplementation on Homocysteine Levels in Adolescent Epileptics Taking Antiepileptic Drugs: A Single Blind Randomized Controlled Clinical Trial

**DOI:** 10.1177/0972753120925560

**Published:** 2020-05-17

**Authors:** Uma A. Bhosale, Radha Yegnanarayan, Akhil Agrawal, Ashwini Patil

**Affiliations:** 1 Deptartment of Pharmacology, SKNMC, Narhe (Ambegaon), Pune, Maharashtra, India.

**Keywords:** Adolescent, AEDs, hyperhomocysteinemia, CVD, folic acid

## Abstract

Background: Epilepsy is a chronic medical condition that requires long-term therapy with antiepileptic drugs (AEDs). However, long-term employment of AEDs may lead to the onset of hyperhomocysteinemia, which has been found to modulate imperative metabolic mechanisms and induce cardiovascular disorders (CVDs). Therefore, adolescent population that have been diagnosed with epilepsy and utilize AEDs are among the most vulnerable, exhibiting higher risks of developing CVDs.

Purpose: The present study was designed to explore the effects of folic acid (FA) supplementation on AED-induced hyperhomocysteinemia and CVD risk factors in adolescent epileptics.

Methods: The randomized clinical trial included adolescent epileptics (i.e., 10–19 years of age) of either sex, on antiepileptic therapy for > 6 months with high homocysteine levels (i.e., >10.9 µmol/L). At the time of enrolment, their baseline BP, lipid and homocysteine levels were recorded. Participants were randomly assigned to either treatment or placebo groups and received the respective treatments. At the end of the first month, BP, lipid and homocysteine levels were recorded and compared to determine the effect of FA on these parameters.

Results and conclusion: A significant fall in homocysteine levels was observed with FA supplementation (*P* < 0.05). However, this fall was significantly high in valproic acid treated epileptic patients. In addition, we observed an improvement in high-density lipoprotein levels, a risk factor for CVDs, but the change was statistically insignificant (*P* > 0.05). The study results suggest that FA supplementation in epileptic patients receiving AED therapy may minimize AED-induced hyperhomocysteinemia and other CVD risk factors.

## Introduction

Epilepsy is a group of CNS disorders, a chronic medical condition that requires long-term therapy with antiepileptic drugs (AEDs). However, long-term employment of AEDs may lead to the onset of hyperhomocysteinemia. Thiol-containing amino acid homocysteine is an intermediate product formed during methionine metabolism. With age, the average concentration of homocysteine increases and the range of blood homocysteine concentrations within adolescents range from 4.3 μmol/L to 9.9 μmol/L. Blood homocysteine concentration of greater than 10.9 μmol/L is defined as a hyperhomocysteinemia. Elevated blood homocysteine concentrations, however, are associated with an increased risk for cardiovascular disorders (CVDs).^1^ Since the epileptic adolescents are bound to consume AEDs for a longer period of time due to their young age, in comparison to adult populations, this warrants an early intervention to abate hyperhomocysteinemia and its potential to induce CVDs.^2^

**Figure 1.**
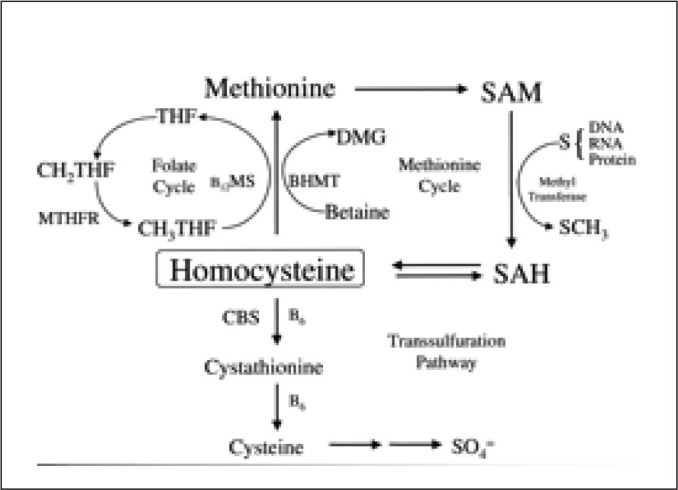
Homocysteine Metabolism Pathway

**Source:** The author.

The re-methylation pathway recycles homocysteine back to methionine and requires vitamin B12 and folic acid (FA) as cofactors (Figure 1).^3^

Asian Indian adolescents are genetically more exposed to CVD risks; AED therapy is an additional risk for developing future CVDs due to folate deficiency leading to homocysteine elevation. It has been observed that homocysteine itself has got epileptogenic potential and can cause the risk developing refractory epilepsy.^4^

Current scientific literature though highlights the role of vitamin B12 in the regulation of blood homocysteine levels; there has been very little research on the implication of FA supplementation on hyperhomocysteinemia, along with AED supplementation for the treatment of epilepsy.^5, 6^ A few studies have reported that there is negative correlation between hyperhomocysteinemia and low FA levels in patients on AEDs.^7^ At the same time, few others have reported effectiveness of FA supplementation to normalize the homocysteine levels.^8^ Therefore, the current study was conducted to study the effects of FA supplementation on homocysteine levels and hyperhomocysteinemia-induced CVD risk factors, including BP and blood lipid levels in adolescent epileptics taking AEDs.

## Materials and Methods^9^

### Study Design

This single-blind two-arm parallel-group randomized controlled clinical trial included adolescent epileptics (n = 42) of either sex with an age range between 10 years and 19 years^10, 11^ taking AEDs for > 6 months. Sample size was calculated by using OpenEpi statistical software expecting a standard deviation (SD) of 2 at an α-error of 5%, power of 80 and mean difference of homocysteine value of 2 µmol/L. In experimental and control groups, patients were allocated in a 2:1 ratio; thus, the minimum required sample size was 24:12.

After getting approval from the Institutional Ethics Committee (Ref. SKNMC No/Ethics/App/229/2014) and requisite informed consent/assent from parents/relatives of patients with high homocysteine levels, i.e., > 10.9 mmol/L (normal homocysteine levels are 4.3–9.9 mmol/L for male and 3.3–7.2 mmol/L for female adolescent and a high homocysteine concentration is defined as at least 11.4 mmol/L for male and at least 10.4 mmol/L for female; gender mean of high homocysteine concentration is 10.9 mmol/L),^12^ were randomly assigned to placebo and test groups by using a table of random numbers obtained from OpenEpi statistical software.

Patients with diabetes, IHD, stroke, malignancy, psychiatric diseases, pregnancy-lactation renal dysfunction, thyroid dysfunction, chronic inflammatory diseases, inborn errors of homocysteine, cobalamin or folate metabolism, or any other condition known to interfere with homocysteine metabolism, receiving vitamin supplements were excluded.

### Homocysteine Assay Method

A fasting blood sample was collected by a standardized procedure. Blood was collected by venipuncture into SST tubes. The serum was separated by centrifugation at 2000 × g for 15 min at 4 °C within 30 min of collection, and samples were immediately stored at －80 °C until analysis. The Diazyme Enzymatic Homocysteine (Hcy) Assay reagent kits were used for the estimation of homocysteine levels by an enzymatic method.

### Statistical Analysis

The data were analysed using OpenEpi (version 2.3); Student’s t-test/ANOVA was used for comparison of the means of continuous variables and normally distributed data. *P* < 0.05 was considered significant.

**Figure:**
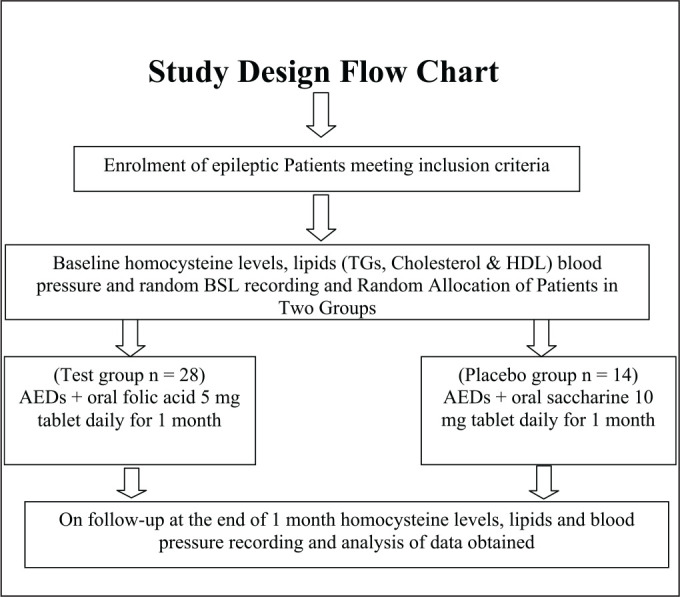


**Table 1.** Baseline Demographic Profile of the Epileptic Patients

**Characteristics** **(N = 14:28)**	**Placebo Group** **(Oral Saccharine)**	**Test Group (Folic Acid 5 mg)**
**Age (mean ± SD)**	16.2 ± 16.8	17.6 ± 12.6
**Sex: male (female)**	09(05)	16(12)
**BMI (mean ± SD)**	23.3 ± 5.3	22.8 ± 2.8

**Source:** The author.

**Table 2.** Effect of Folic Acid Supplementation on Homocysteine Levels of Epileptics

**Characteristics** **N = 14(28)**	**Baseline (mmol/L)** **(Mean ± SD)**	**At 1 Month (mmol/L)** **(Mean ± SD)**
**Placebo group** **(oral saccharine 10 mg)**	27.8 ± 12.9	26.1 ± 12.8
**Test group** **(folic acid 5 mg)**	24.6 ± 6.8	21.7 ± 10.2*

**Source:** The author.

**Note:** N = 42.

*P < 0.05, when compared to baseline levels by Student’s t-test.

**Table 3.** Effect of Folic Acid Supplementation on CVD Risk Factors of Epileptics

**Parameters** **N = 14(28)**	**Placebo Group** **(Oral Saccharine)**		**Test Group** **(Folic Acid 5 mg)**		P **Value**
	**Baseline**	**1 Month**	**Baseline**	**1 Month**	
**SBP (mm of Hg)**	123.9 ± 11.3	124.2 ± 10.8	114.3 ± 4.5	116.3 ± 5.2	> 0.05
**DBP (mm of Hg)**	80.1 ± 9	80.4 ± 9.1	76.8 ± 6.4	76.2 ± 5.6	
**Random BSL (mg/dL)**	111.9 ± 40.3	114 ± 32.6	117.8 ± 10.6	117.2 ± 12.2	
**HDL (mg/dL)**	38.4 ± 7.6	37.3 ± 8.6	39.8 ± 12.1	42.7 ± 16.2	
**TGs (mg/dL)**	137.2 ± 38.8	137.8 ± 32.5	139.3 ± 34	138.1 ± 36	
**Cholesterol (mg/dL)**	186.3 ± 42.8	186.8 ± 38.2	184.2 ± 22.1	183.2 ± 25.3	

**Source:** The author.

**Note:** N = Placebo (test); values are mean ± SD; *P* > 0.05 by ANOVA.

**Abbreviations:** = ± > SBP—systolic blood pressure; DBP—diastolic blood pressure; BSL—blood sugar level; HDL—high-density lipoproteins; TGs—triglycerides.

## Results

**Figure 2.**
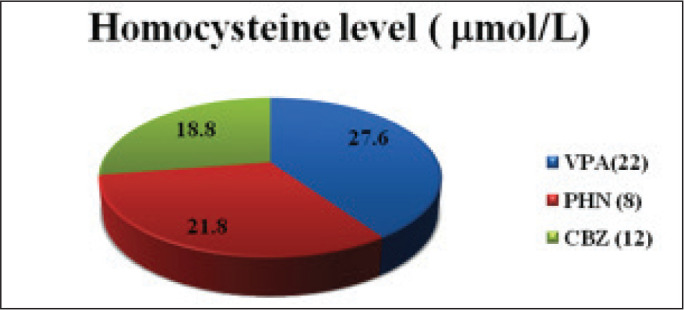
Homocysteine Levels in Epileptics Receiving Various AEDs

**Source:** The author.

**Abbreviation:** VPA—valproic acid; PHN—phenytoin; CBZ—carbamazepine; PHN: phenytoin.

**Note:** A number of patients receiving AED mentioned in parenthesis.

**Figure 3.**
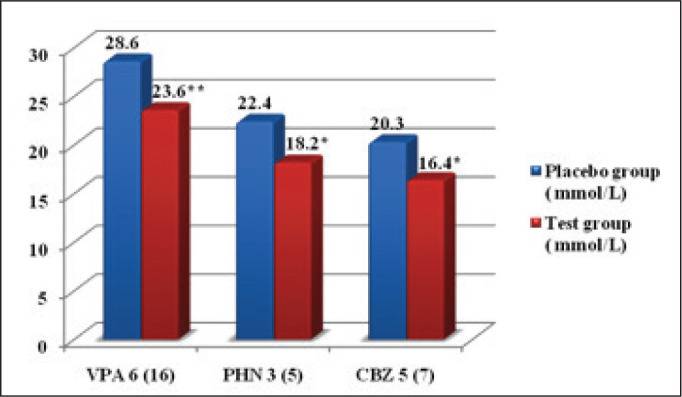
Effect of Folic Acid Supplementation and Placebo on Homocysteine Levels in Various AEDs

**Source:** The author.

**Note:** N = Pplacebo (Ttest); values are Mean±SD. VPA: Valproic Acid, PHN: Phenytoin, CBZ: Carbamazepine.

In this randomized, single-blind and clinical study, we have assessed the effects of FA supplementation on AED-induced hyperhomocysteinemia and other CVD risk factors in adolescent epileptic patients. The demographic profiles of these patients were also studied and are presented in Table 1. There were 25 males and 17 females (mean, 16.2 and 17.6 years of age, respectively) enrolled in this study. The effect of FA supplementation on AED-induced hyperhomocysteinemia showed a significant decrease (*P* < 0.05) in the test group, presented in Table 2. In the present study, the majority of the patients (i.e., 22 participants) were taking valproic acid (VPA), 12 were taking carbamazepine (CBZ) and 8 patients were taking phenytoin (PHN). Homocysteine levels were found to be significantly higher in patients on VPA (Figure 2). However, the greatest reduction in homocysteine levels was found in the patients taking VPA. Overall, the results of the present study revealed a significant fall in homocysteine levels in the test group that was administered FA (Figure 3). Amongst the CVD risk factors that were tested in this study, there was an improvement in test group’s high-density lipoprotein (HDL) levels, but the change was statistically insignificant (Table 3).

## Discussion

**Figure 4.**
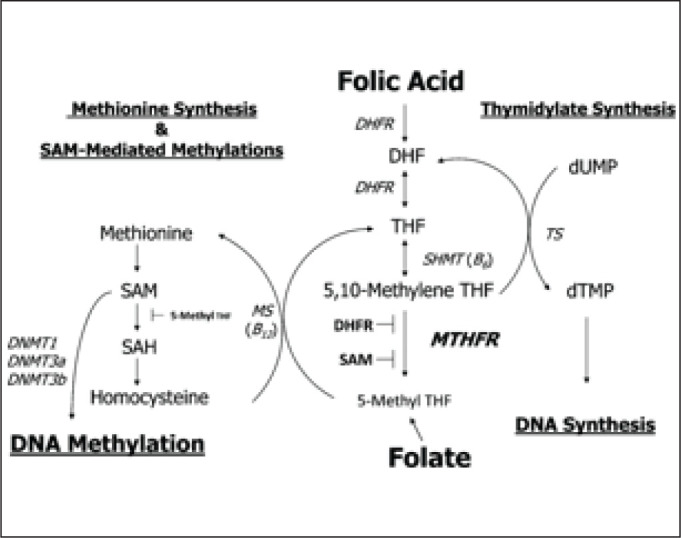
Folate Synthetic Pathway

**Source:** The author.

FA is required for DNA formation where it serves as a carrier of hydroxymethyl and formyl groups. As a derivative from this group, methylterahydrofolate converts homocysteine to methionine, which maintains the blood homocysteine concentration at an appropriate level. However, AEDs have been found to inhibit the conversion of homocysteine to methionine. ^13, 14^ VPA—though a cytochrome P450 enzyme inhibitor, in conjunction with other cytochrome P450 enzyme-inducing AEDs such as CBZ and PHN, has high potential to cause folate deficiency and raised homocysteine levels.^15, 16^ VPA inhibits methionine synthase while other AEDs target methylterahydrofolate reductase inhibiting re-methylation of homocysteine (Figure 1). This leads to hyperhomocysteinemia, which is associated with an increased risk of CVDs in individuals diagnosed with epilepsy. It is believed that homocysteine and its related compounds may have a role as an excitatory agonist on the NMDA subtype of glutamate receptors; epileptic relapse can be discussed on this ground. Hyperhomocysteinemia leads to endothelial cell damage, reduction in the flexibility of vessels and atherosclerosis, and alters the process of haemostasis due to oxidative stress resulting from hypomethylation of homocysteine.^17, 18^ Although prior literature elucidates the effects of FA on homocysteine levels within adults, the results achieved in this study are comparable.^19^ In the present study, FA supplementation showed a significant decrease in blood homocysteine levels within adolescent epileptic patients on various AEDs. FA supplementation replenish levels of folate which facilitate re-methylation of homocysteine into methionine and hence DNA methylation (Figure 4).

*P < 0.05, **P < 0.001 when compared to placebo by sStudent’s’- t-test.

In addition, a fall in homocysteine levels was greatest in VPA-treated patients which is an enzyme inhibitor and this is in contrast with the results of studies which mention that it was significant in enzyme-inducing AEDs such as CBZ and PHN since these AEDs have high potential to cause folate deficiency and raised homocysteine levels.^15, 16^ In these studies, however, only adult epileptic patients were enrolled, while in our study we have enrolled adolescent epileptic patients and majority of them were treated with VPA. Our results are comparable with earlier studies which mention that in children even VPA leads to low folate and high homocysteine levels.^15, 16^ Regarding CVD risk modulation, there were no changes in the measured CVD risk factors following FA administration, not including a minor improvement of HDL within the test group. Although statistically insignificant, more improvement in HDL levels and other CVD risk factors could be induced if the parameters for a study allowed for extended periods of FA supplementation. Therefore, long-term follows-up studies are required to confirm the effects of FA supplementation on CVD risk factors.

## Conclusions

FA supplementation in adolescent epileptics may aid in preventing AED-induced hyperhomocysteinemia, CVDs and epileptic relapse. However, further long-term follow-up studies are required to confirm the effects of FA supplementation on CVD risk factors and prevent epileptic relapse.

## References

[1] Selhub J Miller JW. The pathogenesis of homocysteinemia: interruption of the coordinate regulation by S-adenosylmethionine of the remethylation and transsulfuration of homocysteine. *Am J Clin Nutr* 1991; 55: 131–138. 10.1093/ajcn/55.1.131 1728812

[2] Osganian SK Stampfer MJ Spiegelman D Distribution of and factors associated with serum homocysteine levels in children: Child and Adolescent Trial for Cardiovascular Health. *JAMA* 1999; 281: 1189–1196. 10199428 10.1001/jama.281.13.1189

[3] Hoffer LJ. Homocysteine remethylation and trans-sulfuration. *Metabolism* 2004; 53: 1480–1483. 15536605 10.1016/j.metabol.2004.06.003

[4] Cheng LS Prasad AN Rieder MJ. Relationship between antiepileptic drugs and biological markers affecting long-term cardiovascular function in children and adolescents. *Can J Clin Pharmacol* 2010; 17: e5–46. 20051609

[5] Sato Y Ouchi K Funase Y Relationship between metformin use, vitamin B12 deficiency, hyperhomocysteinemia and vascular complications in patients with type 2 diabetes. *Endocr J* 2013; 60(12): 1275–1280. 24018893 10.1507/endocrj.ej13-0332

[6] Satyanarayana A Balakrishna N Pitla S Status of B-vitamins and homocysteine in diabetic retinopathy: Association with vitamin-B12 deficiency and hyperhomocysteinemia. *PLoS ONE* 2011; 6(11) e26747: 1–7. 10.1371/journal.pone.0026747 PMC3206053 22069468

[7] Coppola G Ingrosso D Operto FF Role of folic acid depletion on homocysteine serum level in children and adolescents with epilepsy and different MTHFR C677T genotypes. *Seizure* 2012; 21: 340–343. 22425007 10.1016/j.seizure.2012.02.011

[8] Linnebank M Moskau S Semmler A Antiepileptic drugs interact with folate and vitamin B12 serum levels. *Ann Neurol* 2011; 69: 352–359. 21246600 10.1002/ana.22229

[9] ClinicalTrials.gov. Efficacy study of folic acid supplementation on homocysteine levels in adolescent epileptics taking antiepileptic drugs: A single blind randomized controlled clinical trial. https://register.clinicaltrials.gov/prs/app/action/adReceipt?draft=true&shovalidate=true&uid=U0002K2H&ts=3&sid=S00059I3&cx=r4b0t4. 10.1177/0972753120925560 PMC7418569 32843833

[10] World Health Organization. *Health topics: Adolescent health*. Geneva: World Health Organization, 2011 Available at: http://www.who.int/topics/adolescent_health/en/

[11] Society. Canadian Paediatric Age limits and adolescents. *Paediatr Child Health* 2003; 8: 577. 20019831 10.1093/pch/8.9.577 PMC2794325

[12] Selhub J Jacques PF Rosenberg IH Serum Total Homocysteine Concentrations in the Third National Health and Nutrition Examination Survey (1991–1994): Population Reference Ranges and Contribution of Vitamin Status to High Serum Concentrations. *Ann Intern Med* 1999; 131(5): 331–339. 10475885 10.7326/0003-4819-131-5-199909070-00003

[13] Paknahad Z Chitsaz A Zadeh AH Effects of common anti-epileptic drugs on the serum levels of homocysteine and folic acid. *Int J Prev Med* Mar 2012; 3(Suppl 1): S186–S190. 22826764 PMC3399294

[14] Schwaninger M Ringleb P Winter R Elevated plasma concentrations of homocysteine in antiepileptic drug treatment. *Epilepsia* 1999; 40: 345–350. 10080517 10.1111/j.1528-1157.1999.tb00716.x

[15] Sener U Zorlu Y Karaguzel O Effects of common anti-epileptic drug monotherapy on serum levels of homocysteine, Vitamin B12, folic acid and Vitamin B6. *Seizure* 2006; 15: 79–85. 16414291 10.1016/j.seizure.2005.11.002

[16] Verrotti A Pascarella R Trotta D Hyperhomocysteinemia in children treated with sodium valproate and carbamazepine. *Epilepsy Res*. 2000; 41: 253–257. 10962216 10.1016/s0920-1211(00)00150-9

[17] Baszczuk A Kopczynski Z. Hyperhomocysteinemia in patients with cardiovascular disease. *Postepy Hig Med Dosw* 2014; 68: 579. 10.5604/17322693.1102340 24864108

[18] Carmel R Jacobsen DW. *Homocysteine in health and disease*. Carmel R Jacobsen DW (eds). Cambridge: Cambridge University Press; 2001; 183–193.

[19] Ono H Sakamoto A Eguchi T Plasma total homocysteine concentrations in epileptic patients taking anticonvulsant. *Metabolism* 1997; 46: 959–962. 9258282 10.1016/s0026-0495(97)90087-1

